# Socioeconomic, lifestyle and biological determinants of cervical screening coverage: Lolland–Falster Health Study, Denmark

**DOI:** 10.1093/eurpub/ckad091

**Published:** 2023-06-09

**Authors:** Milad K Tabatabai, Søren Lophaven, Jeannet Lauenborg, Therese Holmager, Randi Jepsen, Elsebeth Lynge

**Affiliations:** Department of Obstetrics and Gynaecology, Nykøbing Falster Hospital, Nykøbing Falster, Denmark; Omicron Aps, Roskilde, Denmark; Department of Obstetrics and Gynaecology, Nykøbing Falster Hospital, Nykøbing Falster, Denmark; Centre for Epidemiological Research, Nykøbing Falster Hospital, Nykøbing Falster, Denmark; Centre for Epidemiological Research, Nykøbing Falster Hospital, Nykøbing Falster, Denmark; Centre for Epidemiological Research, Nykøbing Falster Hospital, Nykøbing Falster, Denmark

## Abstract

**Background:**

Cervical cancer is preventable. Screening is important for early detection. However, even in high-income countries, coverage is sub-optimal. We identified socioeconomic, lifestyle and biological determinants of cervical screening coverage.

**Methods:**

In Denmark, women aged 23–64 are free of charge personally invited to screening. All cervical cell samples are registered centrally in the Patobank. We linked data from the Lolland–Falster Health Study (LOFUS) with Patobank data. LOFUS was a population-based health survey undertaken in 2016–2020. With logistic regression, coverage defined as ≥1 cervical sample registered within a 6-year period from 2015 to 2020 was compared across levels of risk factors expressed as adjusted odds ratios (aOR) with 95% confidence intervals (CI).

**Results:**

Among 13 406 women of screening aged 23–64 and invited to LOFUS, 72% had ≥1 cervical sample registered. Non-participation in LOFUS was a strong predictor of low coverage; aOR 0.32; 95% CI 0.31–0.36. Among LOFUS participants, education was a strong predictor of coverage in univariate analysis, OR 0.58; 95% CI 0.48–0.71, but this association disappeared in multi-variate analysis, aOR 0.86; 95% CI 0.66–1.10. In multi-variate analysis, predictors of low coverage were high age, living without a partner, retired, current smoker, poor self-rated health, elevated blood pressure and elevated glycated haemoglobin.

**Conclusions:**

Women with low cervical screening coverage had limited contact to healthcare, exemplified by non-participation in LOFUS, and pertinent health and social problems, exemplified by elevated blood pressure and glycated haemoglobin, poor self-rated health, and retirement already in screening age. Structural changes in screening are needed to reach non-screened women.

## Introduction

The combination of human papillomavirus (HPV) vaccination for upcoming generations and cervical screening for generations born in the last century makes cervical cancer a preventable disease. For both interventions, high population coverage is decisive for the effect in the combat against cervical cancer. As one of the tools to eliminate cervical cancer, the WHO recommended that at least 70% of women are screened at age 35 and again at age 45.^1^ A new European Union (EU) proposal, ‘based on the latest available scientific developments and evidence, will support Member States ensuring that 90% of the EU population who qualify for … cervical … screening[s] are offered such screening by 2025’.^2^ In Denmark, all women aged 23–64 have for decades been offered organized cervical screening free of charge and with personal invitation. The stated goal in Denmark is a screening coverage of more than 85%, but the present status is clearly sub-optimal, as only about 70% of the targeted Danish women have been screened within the recommended time intervals.^3^

Low educational level has repeatedly been found to be associated with a low uptake of cervical screening.^4^^,^^5^ In data from the European Health Interview Survey from 2013 to 2015, the odds ratios (OR) for having a cervical smear test within the last 3 years were 0.27 for women with low education and 0.60 for women with intermediate education as compared with women with high education.^6^ Other factors associated with low utilization of cervical smear tests were non-native birth both within and outside the EU, living in a thinly populated area, being never married, being economically inactive and having a low household income. Therefore, efforts to improve cervical screening coverage have focused on improved outreach to socioeconomically disadvantaged women.^7–9^

The evidence base on determinants for use of cervical screening comes mainly from surveys with questionnaire data on socioeconomic and lifestyle factors. To our knowledge, biological determinants of use of cervical screening have not been investigated before, and insight into possible associations between biological factors and use of cervical screening may offer new avenues for intervention.

On this basis, we identified socioeconomic, lifestyle and clinically measured biological determinants of use of cervical screening. We used data from the Lolland–Falster Health Study (LOFUS), a population-based health survey undertaken in 2016–2020 in one of the areas of Denmark with the lowest cervical screening coverage.

## Methods

### Cervical screening in Denmark

In Denmark, women aged 23–49 years are personally invited for screening every third year, and women aged 50–59 years every fifth year. Screening tests are collected by the general practitioners (GPs), and all screening and treatment of detected lesions are free of charge for the women. Up until 2021, liquid-based cytology was used, but from April 2021 onwards half of women aged 30–59 are offered primary HPV screening as part of an implementation trial. All women aged 60–64 years are offered an HPV check-out test.^10^ All samples collected in Denmark are registered centrally in the Danish Pathology Databank (Patobank). Women are invited to screening provided they do not have a cervical cell sample registered in the Patobank within the recommended screening interval. Women not responding to the invitation are sent a first reminder after 3 months, and a second reminder after an additional 6 months.

### Lolland–Falster Health Study

LOFUS is a population-based health survey covering the population from the two municipalities in Lolland–Falster. Data were collected from February 2016 to February 2020.^11^ Lolland–Falster is a rural-provincial area with a population of 103 000 inhabitants. Persons aged 18 and above were randomly selected from the Central Population Register and invited along with all household members to participate in LOFUS. The study included a self-administered questionnaire, a health examination with anthropometric and physiological measurements, and collection of biological samples for same-day analysis and biobanking. In total, 53 000 persons were invited out of whom 19 000 persons participated; representing a participation rate of 36%. Administratively, LOFUS was separated from the cervical screening program as well as from other healthcare services.

### Study design and exposure variables

We included women born 1 January 1953–31 December 1994 and invited to LOFUS. Women were classified by age in the middle of the LOFUS data collection period. For age-classification see Supplementary table S1. Three groups of exposures were considered.

1. Socioeconomic characteristics (age, municipality, citizenship, marital status, education and employment status). Data on age, municipality and citizenship were retrieved from the Central Population Register and therefore available for all women invited to LOFUS. Data on marital status, education and employment were collected from the self-administered questionnaires filled in online by all LOFUS participants before attending the clinical examination.
2. Lifestyle characteristics [smoking, alcohol consumption (≥5 units in sequence) and self-rated health]. These data were also collected from the self-administered questionnaires. Some data were missing. About 4–5% of participants had not answered the questions on marital status, education, employment, smoking and self-rated health, and 17% had not answered the question on alcohol consumption.
3. Biological characteristics [body mass index, blood pressure, pulmonary function and long-term blood sugar (glycated haemoglobin/HbA1c)]. These data were all collected at the clinical examination in a study clinic. For details about the biological data collection, see Supplementary tables S2–S5 and references.^12–15^ While data on body mass index, blood pressure and HbA1c were available for almost all participants, data on pulmonary function were missing for 13% of the participants for whom it had not been possible to undertake a spirometry examination.

### Outcome variables

The outcome variable was having at least one cervical cell sample registered in the Patobank between 1 January 2015 and 31 December 2020; this was called coverage for short. The period was defined to allow all women of screening age to be invited to screening at least once in the time period overlapping the LOFUS data collection. LOFUS and Patobank data were linked using the unique Danish personal identification numbers.

### Statistical analyses

First, we analysed coverage by participation in LOFUS. For LOFUS participants and non-participants, respectively, number of women with at least one cervical cell sample in the study period were divided by number of women in the group. We used logistic regression to estimate the OR of coverage for non-participants in LOFUS compared with participants in LOFUS adjusting (aOR) for the variables age, municipality and citizenship available for all women invited to LOFUS. We included 95% confidence interval (CI).

Second, we analysed coverage in LOFUS participants by the socioeconomic, lifestyle and biological characteristics. We used logistic regression to estimate the univariate OR and multivariate aOR of coverage across levels of each exposure variables including 95% CI and *P* values for differences between the studied exposure level and the reference level. *P* values <0.05 were considered statistically significant. The analysis was undertaken with R version 4.1.0.

### Ethics

Region Zealand’s Ethical Committee on Health Research (SJ-421) approved LOFUS. In accordance with the Declaration of Helsinki, written informed consent was obtained from each participant. Region Zealand approved use of data from the Patobank (R-20067956). The data in this study were anonymized. Access to and handling of data is registered in Region Zealand (REG-144-2020).

## Results

**Figure 1:**
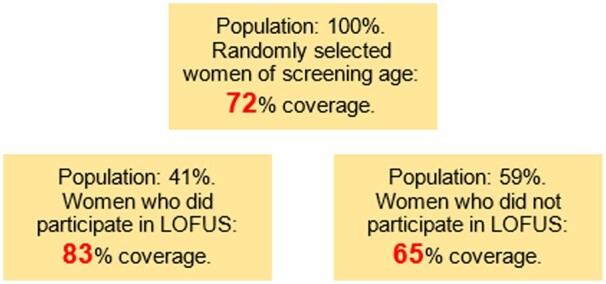
Cervical screening coverage by participation status in the Lolland–Falster Health Study, LOFUS

In total, 13 406 women born from 1 January 1953 to 31 December 1994 were invited to LOFUS, of whom 9691, 72.3%, had at least one cervical cell sample in the study period. Coverage was considerably lower in women who did not participate in LOFUS than in women who participated in LOFUS, 65.4% and 83.4%, respectively (table 1 and figure 1). Adjusted for age, municipality and citizenship, the aOR for coverage was 0.32; 95% CI 0.31–0.36 for non-participants compared with participants.

**Table 1:** Age distribution of women invited to the LOFUS aged 23–64 years by participation in LOFUS (LOFUS±) and by cervical screening coverage (Screen±)

	Population (*n*)	Total			LOFUS+			LOFUS−		
		Screen+ (*n*)	Screen− (*n*)	Coverage	Screen+ (*n*)	Screen− (*n*)	Coverage	Screen+ (*n*)	Screen− (*n*)	Coverage (%)
**All women**	13 406	9691	3715	72.3%	4585	910	83.4%	5106	2805	64.5
**Age (years)^a^**										
23–39	3766	2914	852	77.4%	1020	124	89.2%	1894	728	72.2
40–49	3246	2567	679	79.1%	1219	136	90.0%	1348	543	71.3
50–59	4241	2901	1340	68.4%	1574	374	80.8%	1327	966	57.9
60–64	2153	1309	844	60.8%	772	276	73.7%	537	568	48.6

a Please refer to Supplementary table S1 for definition of age groups.

**Figure 2:**
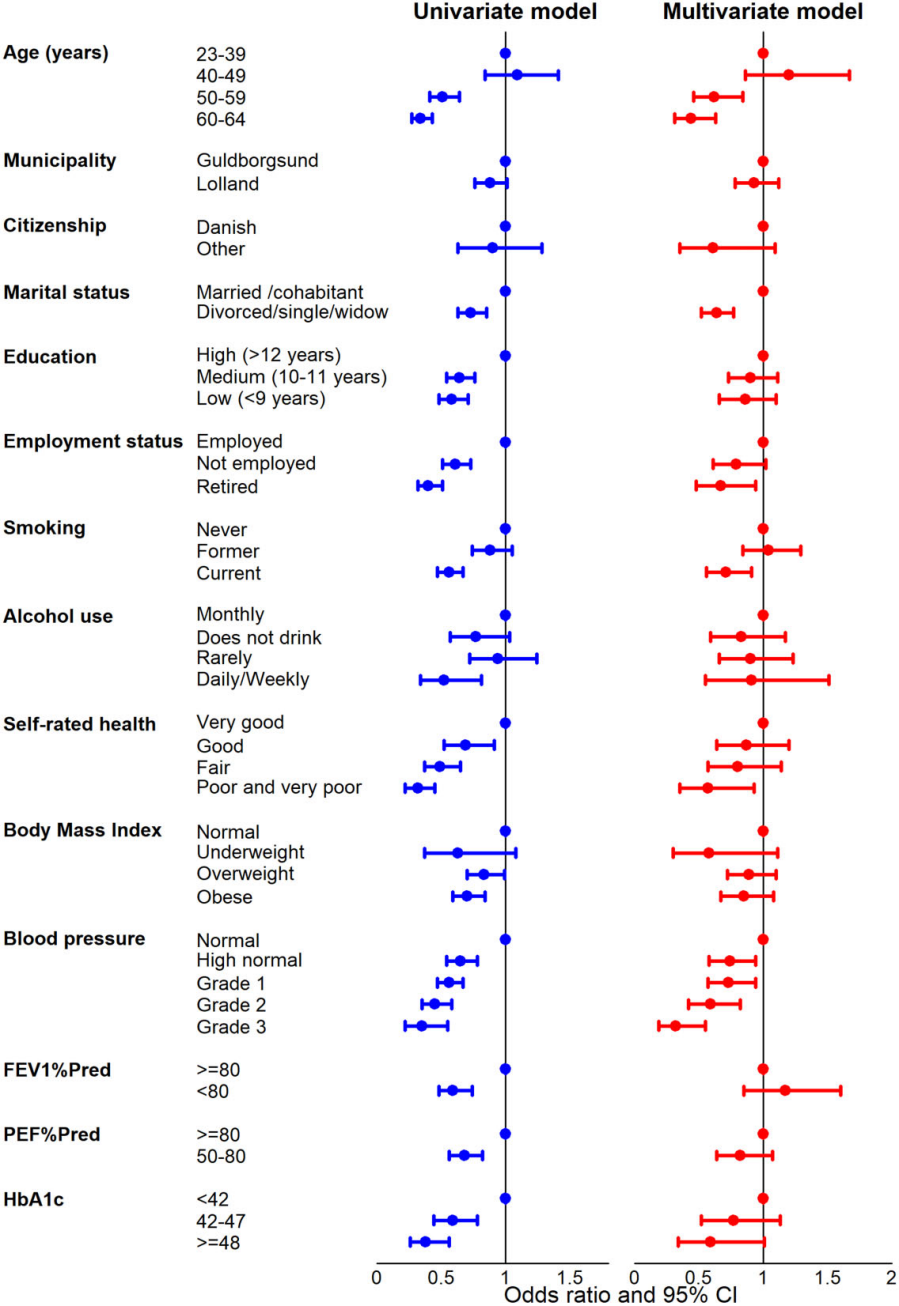
Univariate and mutually aOR and 95% CI of coverage with at least one cervical cell sample by socioeconomic, lifestyle and biological characteristics for women participating in LOFUS

Among women participating in LOFUS, there was no significant difference in coverage between women in age groups 23–39 years and 40–49 years, while coverage was considerably lower in women ≥50 years (figure 2 and Supplementary table S6). In the multi-variate analysis, the OR was 0.62; 95% CI 0.46–0.84 for women aged 50–59 years, and OR 0.44; 95% CI 0.31–0.63 for women aged 60–64 years using women aged 23–39 years as reference. Coverage was nearly similar for women residing in Lolland and Guldborgsund municipalities, and for women with Danish or other citizenship. Marital status as divorced/single/widow was associated with lower coverage than being married/cohabitating; aOR 0.64; 95% CI 0.52–0.77.

In the univariate analysis, education was a strong predictor of coverage. Compared with women with high education, women with low education had an OR of 0.58; 95% CI 0.48–0.71, and women with medium education an OR of 0.64; 95% CI 0.54–0.76. These ORs became insignificant when the other exposure variables were controlled for; aOR 0.86; 95% CI 0.66–1.10 and aOR 0.90; 0.73–1.11, respectively. A somewhat similar pattern was seen for employment status. In the univariate analysis, compared with employed women, not employed women had an OR of 0.61; 0.51–0.73, and retired women an OR of 0.40; 95% CI 0.32–0.51. Only the latter estimate remained statistically significant in the multi-variate analyses, aOR 0.67; 95% CI 0.48–0.94.

Participants in LOFUS who had never smoked or were former smokers had similar coverages of 84–86%, while coverage was lower in current smokers 77.3%; aOR 0.71; 95% CI 0.56–0.91 with reference to never smokers. While monthly or rare drinkers of alcohol had a coverage of 85–86%, the coverage of daily/weekly drinkers was 76.3%, but this difference was statistically insignificant in the multi-variate analysis; aOR 0.91; 95% CI 0.55–1.51. High coverage of 89.2% was seen in women reporting very good self-rated health in contrast to only 72.3% in those reporting poor or very poor health, a difference which remained statistically significant in the multi-variate analysis; aOR 0.57; 95% CI 0.35–0.93.

Coverage was 78.8% in underweight women, 83.0% in overweight, and 80.5% in obese women in contrast to 85.4% in normal weight women, but none of these differences were statistically significant in the multivariate analysis.

Coverage decreased by increasing blood pressure from 87.4% in women with normal blood pressure to 70.8% in women with grade 3 hypertension. This gradient remained statistically significant in the multi-variate analysis. Using women with normal blood pressure as reference, the aOR was 0.74; 95% CI 0.58–0.94 for women with high normal blood pressure, and it decreased to aOR 0.32; 95% CI 0.19–0.55 for women with grade 3 hypertension.

For the three remaining biological risk factors, forced expiratory volume in 1 s as percentage of predicted (FEV1%Pred); peak expiratory flow as percentage of predicted (PEF%Pred); and long-term blood sugar (glycated haemoglobin, HbA1c), coverage was higher in women with favourable values than in women with more problematic values. However, only a HbA1c value ≥48 mmol/mol remained at the borderline of statistically significance in the multivariate analysis, aOR 0.59; 95% CI 0.34–1.01.

## Discussion

### Main findings

Overall, 72% of women of screening age invited to LOFUS, a population-based health study at Lolland–Falster in Denmark, had at least one cervical cell sample taken within the 6-year study period. The strongest determinant for having a sample taken was participation in LOFUS. Controlled for age and the other demographic variables, the odds of having a cell sample registered in women not participating LOFUS was only one-third of that in women participating in LOFUS.

Among LOFUS participants, coverage with cervical cell samples varied across many socioeconomic and lifestyle factors. However, only a few associations remained statistically significant in the multi-variate analysis. Women above the age of 50, living without a partner, being retired, being a current smoker or reporting poor health had low coverage. Most notably, educational level which was a strong predictor of coverage in the univariate analysis became statistically insignificant in the multi-variate analysis.

Our study was possibly the first one to report on coverage by clinically measured, biological characteristics. Approximately half of the women participating in LOFUS had various degrees of elevated blood pressure and they had statistically significantly lower coverage than the other half of women with normal blood pressure. Finally, coverage tended to be low in women with elevated level of glycated haemoglobin.

### Comparison with other studies

In the present study, the overall coverage with at least one cell sample was 72% in women invited to LOFUS. This proportion was higher than the 59% and 63% reported for Lolland and Guldborgsund municipalities, respectively, in the 2021 annual monitoring statistics for cervical screening in Denmark,^3^ but our observation period was longer than the one used in the monitoring statistics.

Inequalities in uptake of cervical screening is a concern both in low- and high-income countries.^7–9^ In Denmark, determinants of coverage in cervical screening were published in 1996. Based on data from 1807 women, the study showed that never-attenders had less frequent contact with their GPs than attenders, were more often nulliparous, and living without a partner.^16^ A Danish nationwide register study from 2014 included 1 052 447 women and found that low coverage was associated with living without a partner, foreign nationality and low education. However, the strongest determinants for low coverage were old age, no contact with dental services OR 0.42 (95% CI 0.42–0.43) and limited contact with GPs OR 0.57 (95% CI 0.56–0.57).^17^ A Danish register study of 476 670 women aged 23–49, invited to screening, and without active op-out, identified risk factors for non-attendance as origin from less developed countries, living without a partner, low education and income, ≥4 children, smoking during pregnancy, ≥2 induced abortions, obesity, intoxicant abuse, and schizophrenia and other psychoses. An association between comorbidity and non-attendance became statistically insignificant when socioeconomic factors were controlled for.^18^ Another Danish study of 14 271 women aged below 46 and having responded to a questionnaire on lifestyle factors, showed obesity, poor self-rated health and daily smoking as determinants for low screening coverage.^19^

In a Swedish register study, determinants of low cervical screening coverage were investigated by comparing long-term non-screened women with women invited to screening and attending within 90 days. Coverage varied considerably across the 21 Swedish counties, and it was low in women born outside Sweden, with low income, being outside the labour force, being on welfare benefit, having a low education and living without a partner.^20^ In Norway, cervical screening coverage was investigated in 12 058 women aged 25–45 years who responded to a questionnaire.^21^ Risk factors for low coverage were being single, reporting excellent self-rated health, current smoking and no wine drinking, no use of oral contraceptives and no use of condoms. The Nordic studies were all cross-sectional, and it is therefore interesting to note that in the English cervical screening programme, which is quite similar to those of Denmark, Norway and Sweden, the disparity in screening coverage between the least and the most deprived areas remained constant from 2007 to 2012.^22^

### Strengths and limitations

The strength of this study was the linkage of LOFUS and Patobank data with unique personal identification numbers, and the completeness of the cervical cell samples data in the Patobank. Moreover, our study included clinically measured, biological variables as possible determinants for screening coverage; to our knowledge, not investigated before.

The most concerning limitation to our study was the low participation rate in LOFUS, namely 41% in women in the age group studied. The strongest risk factor for low screening coverage was non-participation in LOFUS. For the study of risk factors known only for LOFUS participants, this may create a selection bias where it can be problematic to generalize our findings to the entire population. This is a limitation shared with other survey-based studies. Furthermore, data on, e.g. alcohol use were underreported. Out of the 924 women with missing alcohol data, 730 had a cervical cell sample registered, equal to 79.0% and close to that of daily/weekly drinkers.

Finally, coverage was defined as having had at least one cervical cell sample during the observation period of 6 years, and we did not separate out sampling due to primary screening or control for previous abnormal findings, nor did we distinguish between women who actively opted out of screening and women who did not attend after a screening invitation. Finally, we did not exclude hysterectomized women. In the annual monitoring statistics of cervical screening, 4% of women aged 23–64 had opted out due to hysterectomy.^3^

### Public health implications

The experience from northern Europe, including Denmark, is that offering cervical screening free of charge and with personal invitation/reminders does not in itself ensure a universally high screening coverage. Some groups are missed out. In the present study, the strongest determinant of low coverage was non-participation in LOFUS, and in a previous nationwide register-based study, it was limited contact to healthcare as dental services and GPs.^17^

Among participants in questionnaire-based surveys, an inverse association between educational level and coverage has been documented consistently. However, in the present study, this inverse association disappeared when other factors like key biomarkers were controlled for. This could indicate that low coverage was not attributable to poor information as such, but more likely to the women’s focus on more pertinent health and social problems. This interpretation was supported by low coverage among women reporting poor self-rated health, and among retired women given that illness is an eligibility criterium for retirement pension in screening age.

To increase coverage in a setting like the Danish one, the challenge therefore is to reach women with health and social problems and at the same time with limited engagement with health care and/or other health-related initiatives like LOFUS. Alternatives to the present invitation to book an appointment for a clinician-collected sample are therefore warranted.

One possibility is to encourage women and GPs to take a cervical sample when woman is visiting for other reasons. This is already widely practiced in Denmark where almost one-third of cervical samples are taken opportunistically, mostly shortly before or shortly after the scheduled time for screening.^23^ But this is not a solution for women with limited contact to healthcare. A randomized trial in Norway indicated that pre-scheduled screening appointments to women who have failed to participate in screening in the past 4 years would increase overall coverage from 74% to 80%.^24^ In two randomized trials from Sweden, a reminder in the form of a personal telephone call increased coverage.^25^^,^^26^ However, among 4000 women randomized to telephone contact, only 718 were eventually screened, 18% versus 11% in the control group.^26^

Distribution of self-collected cervical cell samples for HPV testing is another possibility.^27^^,^^28^ In one Danish region, 25% of women sent the standard second reminder later attended screening, while this was the case for 38% of women sent instead a self-sampling kit for HPV testing.^28^ This difference would increase overall coverage by approximately 4%.^29^ This procedure is implemented in two of the five Danish regions.^25^ In addition, one region sent self-sampling invitations to under-screened women. Testing kits were returned by 17%, and an additional 14% of the women had a sample taken by a clinician.^30^

All tested interventions showed some improvement in coverage, around 4–6%. Telephone calls and distribution of HPV test kits are expected to be more expensive than pre-scheduled appointments, but pre-scheduled appointments are probably difficult to implement in GP offices.

## Conclusion

In the present study, we investigated coverage by cervical cell samples over a 6-year period in a population-based group of women invited to the LOFUS Health Survey. We found a remarkably large divide with a coverage of 65% in women who did not participate in LOFUS and of 83% in women who participated in LOFUS. This divide of almost 20 percentage points underlined that both the international and Danish goals for universally high screening coverage are very ambitious.

In line with international literature, LOFUS participants with low education had low coverage, but this association disappeared when the other variables including key biomarkers were controlled for. The key message from our study was that to increase screening coverage in the northern European setting, it seems important to reach women with limited use of and engagement in healthcare and with other pertinent health and social problems.

## Supplementary Material

ckad091_Supplementary_Data

## Data Availability

The data underlying this article were provided by Region Zealand by permission. Data will be shared on request to the corresponding author with permission of Region Sjælland.
