# Correction to: Socioeconomic, lifestyle and biological determinants of cervical screening coverage: Lolland–Falster Health Study, Denmark

**DOI:** 10.1093/eurpub/ckad223

**Published:** 2023-12-29

This is a correction to: Milad K Tabatabai, Søren Lophaven, Jeannet Lauenborg, Therese Holmager, Randi Jepsen, Elsebeth Lynge, Socioeconomic, lifestyle and biological determinants of cervical screening coverage: Lolland–Falster Health Study, Denmark, *European Journal of Public Health*, Volume 33, Issue 4, August 2023, Pages 568–573, https://doi.org/10.1093/eurpub/ckad091

In the originally published version of this manuscript, on page 570 “Based on data from 1807” should read “Based on data from 1 807 women”.

The author’s name was also incorrectly presented in the supplementary data associated with this article. “Tabatabar” should read “Tabatabai”.

This error has been corrected online.

