# Supplementary material for: Socioeconomic, lifestyle and biological determinants of cervical screening coverage: Lolland–Falster Health Study, Denmark
Source: Eur J Public Health. 2023 Jun 9;33(4):568–73. doi: 10.1093/eurpub/ckad091 (PMC10393493; doi:10.1093/eurpub/ckad091)
Supplement: ckad091_Supplementary_Data [file ckad091_supplementary_data.pdf]

SUPPLEMENTARY MATERIAL

Tabatabai et al. Socioeconomic, lifestyle, and biological determinants of cervical screening coverage. Lolland-Falster Health Study, Denmark

Supplementary Table S1. Classification of age groups

Screening policy	Age group in years	Year of birth
Below screening age	≤22	1995-1996
Invited every 3rd year	23-39	1978-1994
Invited every 3rd year	40-49	1968-1977
Invited every 5th year	50-59	1958-1967
Check-out test	60-64	1953-1957
No screening offered	65-69	1948-1952
Screening offered in 2017	≥70	1947

Supplementary Tables S2. Definition of biological exposure variables

Body Mass Index	Body mass index was based in measured height and weight at the clinical examination and calculated as weight in kilograms divided by height in meters squared (kg/m^2), and for descriptive purposes categorized into ‘underweight’ (<18.5), ‘normal’ (18.5-24.9), ‘overweight’ (25.0-29.9), and ‘obese’ (≥30.0).
Blood pressure	Systolic and diastolic blood pressures were measured after five minutes of rest and based on three consecutive digital measurements on the upper left arm (apparatus type Welch Allyn Connex pro BPO 3400). The mean values of the second and third measurements were used in this study (only one measurement was used if the other was missing). For classification of blood pressure, see [12] Supplementary TableS3.
Pulmonary function	Pulmonary function was measured by trained healthcare professionals using the MicroLoop Handheld Spirometer™ and SpiroUSB™ with Spirometry PC Software (CareFusion Corp., USA). Sex, height and ethnic origin (Caucasian or Asian) were entered into the software, and the spirometry was performed in a standing position (if possible) with the use of a nose clip. There was no restriction on behavior or medication prior to the measurement, and bronchodilator was not administered prior to spirometry [13]. We used

	measurements of forced expiratory volume in 1 second as percentage of predicted (FEV1%Pred), and peak expiratory flow as percentage of predicted (PEF%Pred). For classification intervals see Supplementary Table S4 and reference [14].
Blood sample	Non-fasting blood samples were collected in vacutainer blood collection tubes (Becton, Dickinson and Company; Franklin Lakes, NJ, USA) and kept at room temperature until same day analysis at the Department of Clinical Biochemistry at Nykøbing Falster Hospital, accredited by the standard ISO 15189. Long-term blood sugar was measured by glycated hemoglobin, HbA1c. For classification intervals see Supplementary Table S5 and reference [15].

Supplementary Table S3. Classification of blood pressures [11]

**Systolic blood pressure (mmHg)**	**Diastolic blood pressure (mmHg)**					
	<50	50-79	80-84	85-94	95-104	≥105
<100	Low	Normal	High normal	Grade 1	Grade 2	Grade 3
100-124	Normal	Normal	High normal	Grade 1	Grade 2	Grade 3
125-134	High normal	High normal	High normal	Grade 1	Grade 2	Grade 3
135-154	Grade 1	Grade 1	Grade 1	Grade 1	Grade 2	Grade 3
155-174	Grade 2	Grade 2	Grade 2	Grade 2	Grade 2	Grade 3
≥175	Grade 3	Grade 3	Grade 3	Grade 3	Grade 3	Grade 3

Supplementary Table S4. Our modified classification of lung function [13]

Spirometry	Severely low	Moderately low	Mildly low to normal
**FEV1%Pred[b]**	<60%	60-80%	>80%
**PEF%Pred[c]**	<50%	50-80%	>80%

[b] Forced Expiratory Volume in 1 second as percentage of predicted.
[c] Peak Expiratory Flow as percentage of predicted.

Supplementary Table S5. Classification of levels of long-term blood sugar, HBA1c [14]

Classification	HbA1c level (mmol/mol)
Normal	<42
Pre-diabetes	42-47
Diabetes	≥48

Supplementary Table S6. Cervical screening coverage by socioeconomic, lifestyle and biological characteristics in women participating in LOFUS (LOFUS+) and aged 23-64 years.

	Population		LOFUS+			**Univariate**		**Mutually adjusted**[a]	
	n	Proportion	Screen+ (n)	**Screen- (*n*)**	**Cover-age**	**OR**	**P-value**	**aOR**	**P-value**
Socioeconomic characteristics									
Age (years)									
23-39	1144	20.8%	1020	124	89.2%	Ref.	Ref.	Ref.	Ref.
40-49	1355	24.7%	1219	136	90.0%	1.09	0.5129	1.20	0.2723
50-59	1948	35.5%	1574	374	80.8%	0.51	<0.0001	0.62	0.0018
60-64	1048	19.1%	772	276	73.7%	0.34	<0.0001	0.44	<0.0001
Municipality									
Lolland	2133	38.8%	1756	377	82.3%	0.88	0.0769	0.93	0.4565
Guldborgsund	3362	61.2%	2829	533	84.1%	Ref.	Ref.	Ref.	Ref.
Citizenship									
Danish	5273	96.0%	4403	870	83.5%	Ref.	Ref.	Ref.	Ref.
Other	222	4.04%	182	40	82.0%	0.90	0.5510	0.61	0.0943
Marital status									
Married /cohabitant	3529	75.7%	2998	531	84.7%	Ref.	Ref.	Ref.	Ref.
Divorced/single/widow	1682	19.1%	1354	328	82.0%	0.73	<0.0001	0.64	<0.0001
Missing	284	5.2%	233	51					
Education									
Low (<9 years)	1121	20.4%	896	225	79.9%	0.58	<0.0001	0.86	0.2312
Medium (10-11 years)	1889	34.4%	1539	350	81.5%	0.64	<0.0001	0.90	0.3290
High (>12 years)	2219	40.4%	1936	283	87.2%	Ref.	Ref.	Ref.	Ref.

Missing	266	4.8%	214	52					
Employment status									
Employed	3813	69.4%	3280	533	86.0%	Ref.	Ref.	Ref.	Ref.
Not employed	1060	19.3%	837	223	79.0%	0.61	<0.0001	0.79	0.0723
Retired	372	6.8%	265	107	71.2%	0.40	<0.0001	0.67	0.0223
Missing	250	4.5%	203	47					

[a] For all other variables in Supplementary Table S6.

Suppelmentary Table S6 continued. Cervical screening coverage by socioeconomic, lifestyle and biological characteristics in women participating in LOFUS (LOFUS+) and aged 23-64 years.

	Population		LOFUS+			Univariate		Mutually adjusted[a]	
	n	Proportion	Screen+ (*n*)	Screen- (*n*)	Coverage	OR	P-value	aOR	P-value
**Lifestyle characteristics**									
**Smoking**									
Never	2457	44.7%	2111	346	85.9%	Ref.	Ref.	Ref.	Ref.
Former	1668	30.4%	1406	262	84.3%	0.88	0.1486	1.04	0.6975
Current	1115	20.3%	862	253	77.3%	0.56	<0.0001	0.71	0.0057
Missing	255	4.6%	206	49					
**Alcohol use**									
Does not drink	1071	19.5%	883	188	82.4%	0.77	0.0810	0.83	0.2903
Rarely	2831	51.5%	2414	417	85.3%	0.94	0.6828	0.90	0.5058
Monthly	492	9.0%	423	69	86.0%	Ref.	Ref.	Ref.	Ref.
Daily/Weekly	177	3.2%	135	42	76.3%	0.52	0.0032	0.91	0.7190
Missing	924	16.8%	730	194					
**Body Mass Index**									
Underweight	85	1.5%	67	18	78.8%	0.63	0.0945	0.58	0.1012
Normal	2164	39.4%	1849	315	85.4%	Ref.	Ref.	Ref.	Ref.
Overweight	1720	31.3%	1427	293	83.0%	0.83	0.0349	0.89	0.2902
Obese	1408	25.6%	1133	275	80.5%	0.70	<0.0001	0.85	0.1779
Missing	118	2.1%	109	9					
**Self-rated health**									
Very good	594	10.8%	530	64	89.2%	Ref.	Ref.	Ref.	Ref.
Good	2948	53.6%	2510	438	85.1%	0.69	0.0096	0.87	0.3952
Fair	1426	26.0%	1144	282	80.2%	0.49	<0.0001	0.80	0.2137
Poor and very poor	289	5.3%	209	80	72.3%	0.32	<0.0001	0.57	0.0255
Missing	238	4.3%	192	46					

[a] For all other variables in Supplementary Table S6.

Supplementary Table S6 continued. Cervical screening coverage by socioeconomic, lifestyle and biological characteristics in women participating in LOFUS (LOFUS+) and aged 23-64 years.

	Population		LOFUS+			Univariate		Mutually adjusted[a]	
	n	Proportion	Screen+(*n*)	Screen-(*n*)	Coverage	OR	P-value	aOR	P-value
**Biological characteristics**									
**Body Mass Index**									
Underweight	85	1.5%	67	18	78.8%	0.63	0.0945	0.58	0.1012
Normal	2164	39.4%	1849	315	85.4%	Ref.	Ref.	Ref.	Ref.
Overweight	1720	31.3%	1427	293	83.0%	0.83	0.0349	0.89	0.2902
Obese	1408	25.6%	1133	275	80.5%	0.70	<0.0001	0.85	0.1779
Missing	118	2.1%	109	9					
**Blood Pressure[b]**									
Normal	2617	47.6%	2288	329	87.4%	Ref.	Ref.	Ref.	Ref.
High normal	1187	21.6%	972	215	81.9%	0.65	<0.0001	0.74	0.0131
Grade 1	1181	21.5%	940	241	79.6%	0.56	<0.0001	0.73	0.0143
Grade 2	394	7.2%	298	96	75.6%	0.45	<0.0001	0.59	0.0019
Grade 3	96	1.7%	68	28	70.8%	0.35	<0.0001	0.32	<0.0001
Missing	20	0.4%	19	1					
**FEV1%Pred[b]**									
≥80	4263	77.6%	3591	672	84.2%	Ref.	Ref.	Ref.	Ref.
<80	525	9.6%	399	126	76.0%	0.59	<0.0001	1.17	0.3368
Missing	707	12.9%	595	112					
**PEF%Pred[b]**									
≥80	3962	72.1%	3341	621	84.3%	Ref.	Ref.	Ref.	Ref.
50-80	825	15.0%	648	177	78.5%	0.68	<0.0001	0.82	0.1411
Missing	708	12.9%	596	112					
**HbA1c[b] (mmol/mol)**									
<42	5025	91.4%	4234	791	84.3%	Ref.	Ref.	Ref.	Ref.
42-47	274	5.0%	208	66	75.9%	0.59	0.0003	0.77	0.1868
≥48	121	2.2%	81	40	66.9%	0.38	<0.0001	0.59	0.0558
Missing	75	1.4%	62	13					

[a] For all other variables in Supplementary Table S6.

[b] Please refer to Supplementary Tables S2, S3, S4, S5 for definitions of measurements
